# Complex Neurological Sequelae: Axonal Guillain-Barré Syndrome Post COVID-19 in a Young Patient

**DOI:** 10.7759/cureus.67213

**Published:** 2024-08-19

**Authors:** Anam Umar, Amber E Faquih, Bilal Jawed, Muhammad Bilal

**Affiliations:** 1 Internal Medicine, Ascension St. Vincent's Birmingham, Birmingham, USA; 2 Infectious Diseases, University of Alabama at Birmingham, Birmingham, USA; 3 Internal Medicine, Jinnah Postgraduate Medical Centre, Karachi, PAK; 4 Internal Medicine, Ascension St. Vincent’s Birmingham, Birmingham, USA

**Keywords:** guillain-barré syndrome (gbs), acute inflammatory demyelinating polyradiculoneuropathy, axonal guillain-barré syndrome, covid-19, post covid-19 complications

## Abstract

Guillain-Barré syndrome (GBS) encompasses a spectrum of immune-mediated neuropathies, with axonal GBS representing a less common yet often severe subtype. This variant directly damages peripheral nerve axons, resulting in rapid and profound muscle weakness and sensory deficits. Axonal GBS has similar clinical features to the demyelinating form but is generally more severe with a less favorable prognosis. Here, we present a case of axonal GBS in a 46-year-old female following a mild COVID-19 infection, highlighting the diagnostic challenges and the importance of tailored therapeutic approaches and multidisciplinary care in managing this condition.

## Introduction

First described in Wuhan, China, in late 2019, COVID-19 is caused by the new coronavirus structural variant SARS-CoV-2. It spread worldwide, leading to a pandemic that distressed millions of people [1]. The virus spreads through respiratory droplets, impairing various extents from gentle respiratory ailments to serious pneumonia and acute respiratory distress syndrome. On March 11, 2020, the World Health Organization declared COVID-19 a pandemic, marking the starting point of wide implementations of public health measures and thorough research on vaccine and treatment development [2].

Common symptoms included fever, cough, and dyspnea. Atypical but true manifestations are gastrointestinal symptoms and loss of taste or smell, skin manifestations, and neurologic, renal, and reproductive problems reported in COVID-19 patients [3].

Guillain-Barré syndrome (GBS) has been seen in about 0.22% of patients with COVID-19. It presents with lower limb weakness and paresthesia about 5-10 days after the onset of symptoms and may even precede respiratory manifestations. This atypical presentation calls for recognition to diagnose and treat promptly in COVID-19 patients with acute neurological symptoms [4,5]. The outcome of GBS in COVID-19 varies based on timely management. Patients may recover quickly or have lasting deficits requiring extended rehabilitation. Raising awareness about GBS in COVID-19 is crucial for prompt interventions and better results.

## Case presentation

A 46-year-old female with a known history of celiac disease, fibromyalgia, migraines, and depression presented to the emergency room with a complaint of progressive generalized weakness. She reported noticing tingling and numbness in her hands three weeks before this presentation, which eventually progressed to involve the upper limbs and then the lower limbs bilaterally. She reported the progression of this weakness to the point that she stumbled and injured her left ankle two days before admission. Additionally, she had experienced bladder hesitancy but denied swallowing difficulties, double vision, or significant neck or back pain. Notably, she had a mild case of COVID-19 two weeks prior, with symptoms including cough and nasal drainage, which had not required hospitalization.

**Figure 1:**
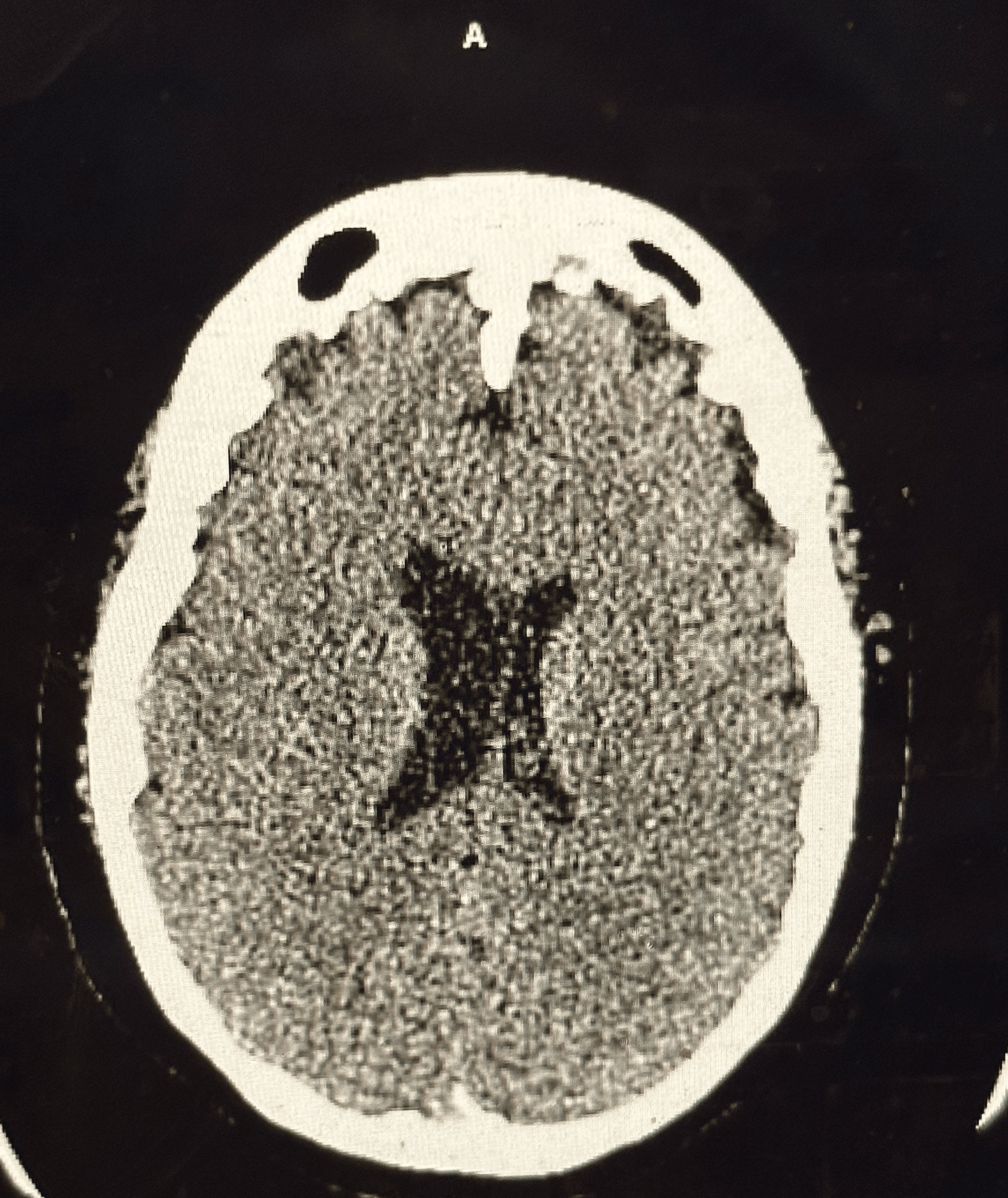
CT scan of head or brain without contrast This image shows no evidence of acute abnormality as a cause of generalized weakness, tingling, and numbness. Ventricles and sulci are normal in size and shape. Ventricular asymmetry is noted, which is a normal variant.

**Figure 2:**
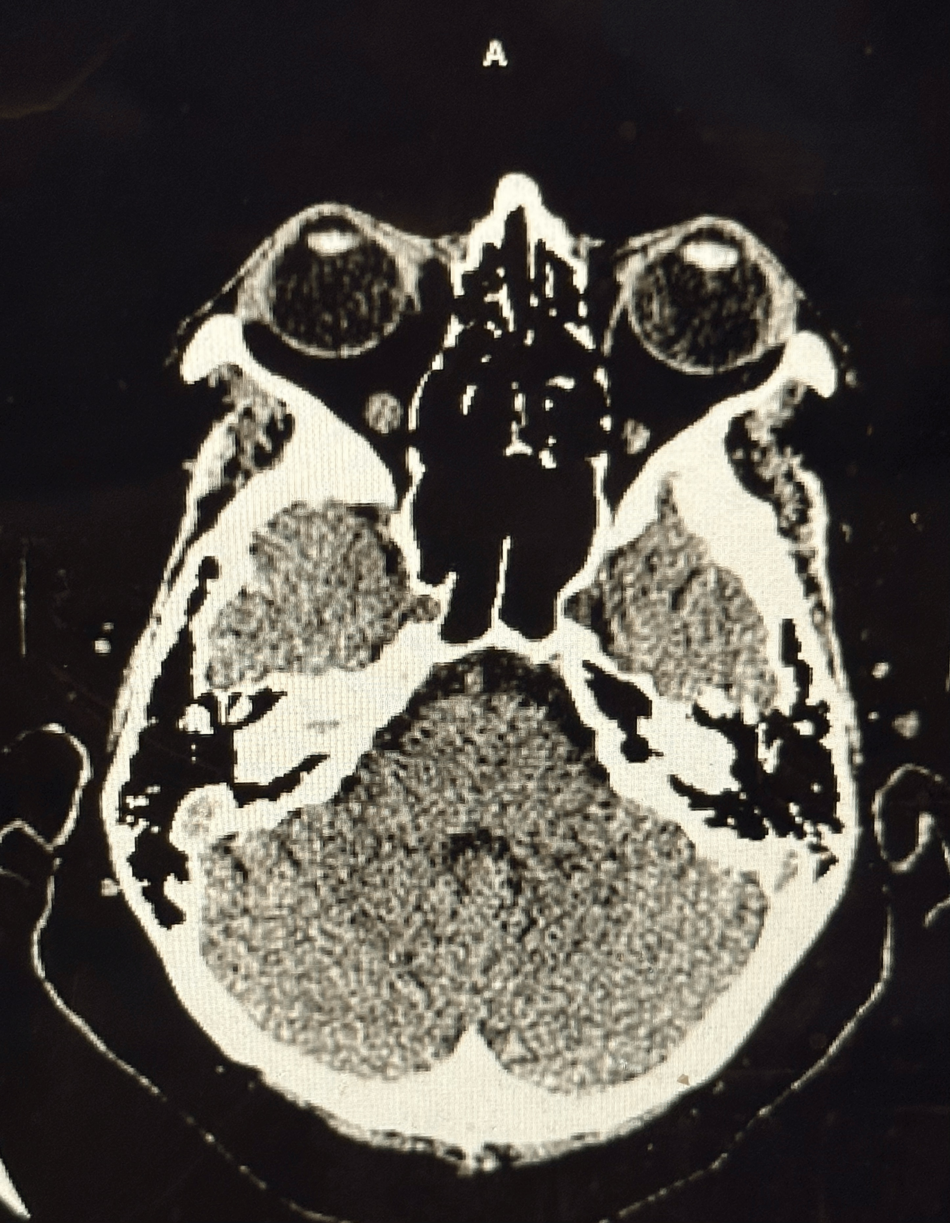
CT scan of the head or brain without contrast This image demonstrates no evidence of acute abnormalities as the cause of generalized weakness, tingling, and numbness.

**Figure 3:**
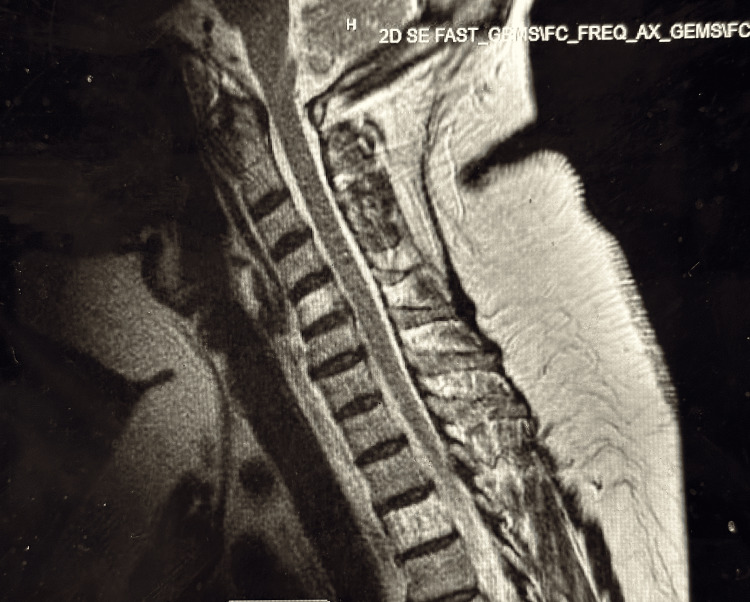
MRI spine cervical There is no evidence of acute abnormality. There is no evidence of spinal cord compression or intrinsic spinal cord abnormality. Mild disc desiccation and disc bulging are present throughout the cervical spine, with no evidence of focal disc herniation or nerve root compression. The cranio-cervical junction is unremarkable. Paraspinal soft tissues do not show any significant abnormality.

Clinical examination on presentation revealed a somewhat atypical presentation, with fair truncal strength but decreasing strength in the distal arms and legs. Upon admission, vital signs were stable, and laboratory investigations showed slight leukocytosis and mildly elevated levels of creatinine, glucose, and liver enzymes. Other biochemical parameters were within normal limits. Imaging studies, including CT scans (Figures 1, 2) and MRI (Figure 3) did not reveal acute abnormalities, but an abnormal nerve conduction study indicated severe polyneuropathy with mixed demyelinating and axonal features.

Lumbar puncture revealed slightly elevated protein levels, suggesting spinal fluid abnormalities (Table 1). Based on these findings, the patient was diagnosed with axonal Guillain-Barré syndrome (GBS) with an atypical presentation. A nerve conduction study (NCV) performed during the hospital stay revealed abnormal nerve conduction velocity in both the upper and lower extremities, indicating severe polyneuropathy with a combination of demyelinating and axonal features. The patient received treatment with IV immunoglobulin (IVIG) at a dose of 400 mg/kg/day for five days, along with physical therapy. Additionally, efforts were made to address comorbidities, vitamin deficiencies, and a urinary tract infection. During IVIG treatment, there was no progression of symptoms, and some improvement in hand weakness was observed. However, after the completion of the treatment, the patient experienced worsened weakness and cold sensations in the hands and feet. Subsequently, the patient received two doses of IVIG at 20 g/dose, which did not improve the worsening symptoms. Therefore, the treatment approach was transitioned to plasmapheresis with a planned total of six sessions. Despite these efforts, the patient showed only slight improvement and continued to require assistance with mobility and daily activities. After discharge, the patient was recommended for multidisciplinary rehabilitation to support optimal functional recovery and eventual independence.

**Table 1:** Lumbar puncture: cerebrospinal fluid analysis The table shows abnormal results with the mention of normal ranges.

Parameter	Result
Color	Colorless (normal)
Clarity	Clear (normal)
Red blood cells (cells/mcL)	44 (normal <=0)
Xanthrochromia	Negative
Glucose (mg/dL)	97 (normal range 40-70)
Protein (mg/dL)	45 (normal range 15-40)
CSF fluid culture and smear	No growth

This case underscored the complexity and challenges associated with GBS, particularly when presenting with unusual clinical features. Continued monitoring and comprehensive management will be essential to achieve optimal outcomes for the patient's ongoing care and rehabilitation.

## Discussion

GBS is an immune-mediated disorder related to the peripheral nervous system and typically presents with weakness reduction or loss of reflexes in the limbs. Epidemiological data estimate that the general population experiences GBS at a rate of 0.8 to 1.9 cases per 100,000 people per year [6]. Approximately two-thirds of GBS cases are due to multiple infections, especially upper respiratory infections [7]. Commonly known infectious agents leading to GBS are Campylobacter jejuni, Haemophilus influenza virus, Cytomegalovirus, enteroviruses, herpes simplex virus, and human immunodeficiency virus (HIV). It is reported that in 2019-2020, there was a five times increase in GBS cases. In many of these reports, there was a clear link between COVID-19 and GBS [8]. Symptoms of GBS-induced polyneuropathy were reported to occur during or after the emergence of COVID-19 symptoms. This observation was supported by a study conducted by Sedaghat et al. and Ottaviani et al. where patients reported symptoms of GBS before, during, and after the COVID-19 symptoms [9,10].

The pathophysiology of COVID-19 causing GBS involves SARS-CoV-2 using angiotensin-converting enzyme 2 (ACE-2) receptors to enter host cells, affecting the lungs, gastrointestinal tract, cardiomyocytes, urothelial cells, and proximal tubular cells [11]. Neurons and glial cells also express these receptors, making the brain vulnerable to SARS-CoV-2, potentially leading to stroke, encephalopathies, and GBS. The virus binding to ACE-2 receptors can trigger cytokine storm production and blood-brain barrier breakdown, resulting in direct nervous tissue damage and an immune response activation. Antibodies produced against the virus may cross-react with gangliosides, causing autoimmune destruction of myelin sheaths or axons. Fantini et al. observed interactions between SARS-CoV-2 spike saccharides and myelin sheaths or axon gangliosides [12].

GBS has demyelinating or axonal subtypes, including acute inflammatory demyelinating polyneuropathy (AIDP), acute motor axonal neuropathy (AMAN), acute motor sensory axonal neuropathy (AMSAN), Miller Fisher (MF) syndrome, and prototypic Bickerstaff encephalopathy. As discussed above GBS typically presents with lower extremity weakness and paresthesia. The two most common types are AMAN and AIDP; AMAN usually causes weakness in all four limbs while AIDP leads to weakness and loss of motor and sensory function [13].

GBS in COVID-19 patients is more prevalent in males over 50. Onset occurs 3-24 days post-infection, with symptoms like facial diplegia with distal weakness, pure sensory or motor variants, MF syndrome, and ataxic variants. Some studies reported facial palsy though this was not widely supported. The demyelinating subtype was the most common, and ICU admissions were higher, with over 30% needing mechanical ventilation compared to non-COVID GBS cases [14]. About 70-85% of patients responded well to treatment. ICU admission rates were higher due to lung injury and systemic involvement from SARS-CoV-2. The AMSAN variant has a slower recovery and higher chance of dysautonomia than AMAN, as macrophages invade the space between Schwann cells and the axon, leaving the myelin sheath intact [15].

Axonal GBS, often linked to Campylobacter jejuni, is most common in Chinese and Asian populations. Its clinical features are similar to classical GBS (AIDP) but with a more severe course, including frequent respiratory involvement, ventilator dependence, cranial neuropathy, prolonged recovery, and significant residual effects [16]. Unlike the natural remyelination in peripheral nerves, axonal damage in this variant leads to poor prognosis [7]. Michel-Chávez et al. noted that axonal GBS (AMSAN/AMAN) patients often require mechanical ventilation [17]. Diagnosing GBS involves CSF analysis showing albuminocytologic dissociation, nerve conduction studies (NCS), and electromyography (EMG). Treatment of GBS is focused on supportive care until there is an indication for immunomodulatory therapy. Immunomodulatory therapy is suggested for severe muscle weakness, rapidly progressing symptoms or symptoms not improving. Usually, it should be started within four weeks of the onset of symptoms and the variant of GBS [18]. There are two options for immunotherapy: plasma exchange and IVIG. Patients with axonal GBS are treated with a course of IVIG and plasmapheresis, just like in this patient [19].

## Conclusions

In conclusion, the case of this 46-year-old female with axonal Guillain-Barré Syndrome (GBS) underscores the diagnostic challenges and complexities associated with this condition, especially when presenting with atypical features. Despite the initiation of therapeutic interventions including IV Immunoglobulin (IVIG) and plasmapheresis, as well as the correction of comorbidities, the patient exhibited only mild improvement in symptoms and continued to require assistance with daily activities. Multidisciplinary rehabilitation was recommended to optimize functional recovery and facilitate independence in activities of daily living post-discharge.

This case highlights the need for continued monitoring and comprehensive management in patients with GBS, particularly in those with unusual clinical presentations. Further research and clinical experience are necessary to better understand and effectively manage this challenging neurological condition.

## References

[1] (2024). WHO Director-General's opening remarks at the media briefing on COVID-19. https://www.who.int/dg/speeches/detail/who-director-general-s-opening-remarks-at-the-media-briefing-on-covid-19---11-march-2020.

[2] (2024). COVID-19 overview and infection prevention and control priorities in non-US healthcare settings. https://archive.cdc.gov/#/details?url=https://www.cdc.gov/coronavirus/2019-ncov/hcp/non-us-settings/overview/index.html.

[3] Guan WJ, Ni ZY, Hu Y (2020). Clinical characteristics of coronavirus disease 2019 in China. N Engl J Med.

[4] Jayasekara D, SeneviratneS SeneviratneS, Jayasekara A, De Zoysa I (2020). Atypical presentations of COVID-19. Adv Infect Dis.

[5] Abobaker A, Raba AA, Alzwi A (2020). Extrapulmonary and atypical clinical presentations of COVID-19. J Med Virol.

[6] Sejvar JJ, Baughman AL, Wise M, Morgan OW (2011). Population incidence of Guillain-Barré syndrome: a systematic review and meta-analysis. Neuroepidemiology.

[7] Willison HJ, Jacobs BC, van Doorn PA (2016). Guillain-Barré syndrome. Lancet.

[8] Gigli GL, Bax F, Marini A, Pellitteri G, Scalise A, Surcinelli A, Valente M (2021). Guillain-Barré syndrome in the COVID-19 era: just an occasional cluster?. J Neurol.

[9] Sedaghat Z, Karimi N (2020). Guillain Barre syndrome associated with COVID-19 infection: a case report. J Clin Neurosci.

[10] Ottaviani D, Boso F, Tranquillini E (2020). Early Guillain-Barré syndrome in coronavirus disease 2019 (COVID-19): a case report from an Italian COVID-hospital. Neurol Sci.

[11] Aleem A, Akbar Samad AB, Vaqar S (2023). Emerging Variants of SARS-CoV-2 and Novel Therapeutics Against Coronavirus (COVID-19). In: StatPearls. Treasure Island (FL): StatPearls Publishing.

[12] Fantini J, Di Scala C, Chahinian H, Yahi N (2020). Structural and molecular modelling studies reveal a new mechanism of action of chloroquine and hydroxychloroquine against SARS-CoV-2 infection. Int J Antimicrob Agents.

[13] Patnaik UJ (2021). Review article on COVID-19 and Guillain-Barré syndrome. Front Biosci (Schol Ed).

[14] Palaiodimou L, Stefanou MI, Katsanos AH (2021). Prevalence, clinical characteristics and outcomes of Guillain-Barré syndrome spectrum associated with COVID-19: a systematic review and meta-analysis. Eur J Neurol.

[15] Abu-Rumeileh S, Abdelhak A, Foschi M, Tumani H, Otto M (2021). Guillain-Barré syndrome spectrum associated with COVID-19: an up-to-date systematic review of 73 cases. J Neurol.

[16] Yuki N, Kuwabara S, Koga M, Hirata K (1999). Acute motor axonal neuropathy and acute motor-sensory axonal neuropathy share a common immunological profile. J Neurol Sci.

[17] Michel-Chávez A, Chiquete E, Gulías-Herrero A (2023). Predictors of mechanical ventilation in Guillain-Barré syndrome with axonal subtypes. Can J Neurol Sci.

[18] Donofrio PD (2017). Guillain-Barré syndrome. Continuum (Minneap Minn).

[19] Zaki HA, Iftikhar H, Najam M (2023). Plasma exchange (PE) versus intravenous immunoglobulin (IVIG) for the treatment of Guillain-Barré syndrome (GBS) in patients with severe symptoms: a systematic review and meta-analysis. eNeurologicalSci.

